# Rape Victims’ Perceptions of Quality of Encounters With the Swedish Police

**DOI:** 10.1177/10778012241243055

**Published:** 2024-04-07

**Authors:** Lisa Rudolfsson, Laura Hammond, Christina Björklund

**Affiliations:** 1 Institute of Environmental Medicine (IMM), 27106 Karolinska Institute, Stockholm, Sweden; 2 Faculty of Business, Law, and Social Sciences, 1725 Birmingham City University, Birmingham, UK

**Keywords:** justice, police, rape victims, questionnaire study

## Abstract

This study focused on raped women's perceptions of their encounters with Swedish police, with a specific focus on quality of encounters, trust, questions asked during police interviews, and perceptions of justice. One hundred and six rape victims, 74 of whom had reported to the police, answered a web-based questionnaire. Results show that where officers explained their line of questioning the perceived intrusiveness of the questioning was lower, as was the level of perceived victim-blaming. Higher quality police encounters were associated with higher trust in the legal system and in police work, and with higher levels of received justice. Findings highlight the importance of trauma-informed policing as a response to sexual assault and provide insights which might help inform policy and practice developments, both in Sweden and more generally.

## Introduction

For many victims, the legal process both starts and ends with their encounters with the police. Consequently, the focus of this study was on raped women's perceptions of encounters with Swedish Police.

### Rape Victims and Police

Rape is associated with victims’ psychological trauma; in particular, with victims’ risk of experiencing anxiety, depression, and posttraumatic stress disorder (PTSD; Maercker et al., 2018; Pegram & Abbey, 2019). Other long-term negative psychological consequences can manifest in victims’ increased fear and worry, self-criticism, self-injury, sexual problems, shame, and guilt (Basile et al., 2021; Bhuptani, 2021; Kaukinen & DeMaris, 2009). Victims’ reactions, however, are complex and depend on individual factors (e.g., prior victimization; Nishith et al., 2000) as well as on situational factors (e.g., the use of physical violence and relation to the perpetrator; Pegram & Abbey, 2019; Ullman et al., 2006). Victims’ reactions have also been linked to the reactions of others. For example, myths and stereotypes about rape and victims often result in victim blaming and stigma (Parratt & Pina, 2017; Ullman & Peter-Hagene, 2014).

Victims have reported that being treated with dignity and respect by police is equally important to them as the legal outcome of their case (Jordan, 2015). However, previous studies describe victims’ anticipation of negative treatment when reporting to the police (Lorenz et al., 2021; Shaw et al., 2017). Victims also frequently report negative or poor treatment in their encounters with police (Sleath & Bull, 2017), sometimes describing it as like being raped again (Temkin & Krahe, 2008). As such, negative police interactions can contribute to victims’ distress and result in revictimization (i.e., exacerbate the suffering associated with the trauma of rape; Ballucci & Drakes, 2021; Campbell, 2006).

However, previous research also emphasizes the role of police in facilitating victims’ coping, showing that a positive police response can reduce trauma and improve victims’ quality of life (Barkworth & Murphy, 2016; Elliott et al., 2011). Consequently, the demeanor of the police has important effects on victims’ comfort and trust in the system, and further, on their motivation to proceed with the legal process and cooperate with the police investigation (Hopper et al., 2020).

It is well-established that the complexities of victim's trauma symptoms and needs, and the complexities of rape investigations more generally, demand specialized police responses (Jordan 2011), and previous research stresses the importance of specialized training among police (McKee et al., 2020; Rich, 2019). Training has been found to improve officers’ sensitivity, compassion, and empathy in the treatment of victims (Jordan, 2002). However, in Sweden, no formal education on the treatment of victims is offered, and only one specialized department for sexual crimes exists (Backhans et al., 2021).

### The Swedish Context

In 2022, approximately 24,660 sexual crimes were reported to Swedish police, of which 9,635 were classified as rapes (https://bra.se/statistik/statistik-utifran-brottstyper/valdtakt-och-sexualbrott.html). Both the Swedish National Council for Crime Prevention (BRÅ) and Swedish police officers have previously described sexual crimes as particularly difficult to investigate (Holmberg & Lewenhagen, 2019; Rudolfsson, 2022). Rape investigations are often characterized by conflicting statements and a lack of physical evidence, which makes it hard to meet evidentiary requirements for the case to be brought to court (Swedish Police Authority, 2019). Although Swedish police conduct a preliminary investigation in 94% of reported cases, only approximately 5% of rape charges result in a conviction (Holmberg & Lewenhagen, 2019). It is estimated that 7%–11% of reported rapes in Sweden are cleared, which is comparable to rates in other European countries and in the United States (Holmberg & Lewenhagen, 2020; Spohn & Tellis, 2014).

Since 2018, Swedish legislation is based on lack of consent, classifying all sexual acts where one party has not consented as rape: “Forced intercourse or equally coercive sexual violation of a person who is unwilling or unable to comprehend or consent or who is in a position of dependence on the perpetrator” (Brottsbalken [Swedish Criminal Code], Ch. 6, §1, author translation). As the new legislation classifies more varied forms of sexual abuse as rape (i.e., dropping the former requirement of victims’ physical resistance), the number of convictions for rape in Sweden has increased. However, difficulties in applying the law are still discussed (BRÅ, 2020).

In Sweden, the police are responsible for an alleged crime being recorded, conducting the preliminary investigation, and transferring the case to the Swedish Prosecution Authority who decide on further actions and whether the case should be brought to court (Swedish Police Authority, 2019). Allegations of rape are generally investigated as serious or domestic crimes. However, due to the high number of gang-related shootings in Sweden, previous studies have found rape allegations to be allocated a lower priority and, as a consequence, less experienced officers can be assigned to investigate rape cases (Backhans et al., 2021). In a previous qualitative study (Rudolfsson, 2023), raped women described how meeting with an empathetic officer was a question of luck, where some described comforting meetings and some felt violated once again. The findings highlight victims’ needs for support and information, and a need for improved trauma-informed responses among Swedish police.

### The Current Study: Aim and Research Questions

The focus of the current study was on raped women's perceptions of their encounters with Swedish police. To investigate this, we constructed an online questionnaire that was distributed to women who had been raped. Participants were not selected; instead, they were recruited through posters and hand-outs at support organizations and gynaecological emergency units. The inclusion criteria were based on their experiences of having reported that they had been raped to the police and/or having sought medical care after being raped.

Specific research questions related to the following: *Not reporting to police:* We explored the reasons for victims *not* reporting to the police, and whether specific vulnerabilities (e.g., neuropsychiatric disability, mental ill-health) were associated with participants’ reporting or not reporting to the police. *Quality of encounters with police*: Based on previous studies (i.e., Rudolfsson, 2022, 2023) we hypothesized that participants that were interviewed by police after the latest Swedish rape legislation came into effect (2018) would report a higher quality of police encounter, and that there would be *no* significant differences in reported quality of police encounter between participants that were interviewed by a female or male officer. We explored whether the reported quality of police encounters differed if participants had certain vulnerabilities. *Questions asked during a police interview*: We explored how relevant, empathic, intrusive, and victim-blaming questions during police interviews were perceived to be, and whether questions asked were perceived in more positive ways if the officers had explained their line of questioning. *Trust in the Swedish legal system and in Swedish police work*: We hypothesized that participants’ trust in the legal system and police work would be lower than the trust reported among women in the general population. We explored whether the quality of police encounters was related to trust in the legal system and in police work and whether trust in the legal system and in police work differed between participants who did or did not report. *Justice and its relation to the quality of police encounters*: We explored perceptions of different forms of justice, to what degree participants reported that they had received justice, and whether participants who had reported to the police had a higher perceived level of received justice than participants who did not report. We hypothesized that participants having their cases tried in court and the perceived quality of police encounters would predict participants’ level of perceived justice.

## Method

### Participants

One hundred and six women took part in the study. The oldest participant was 64 years old, and the youngest was 18 years old (*M *= 33.24, *SD *= 10.71). Most participants were born in Sweden (93.4%), and only four participants were born outside Europe. Approximately half (50.9%) of participants reported living in a large-sized city, and most participants (33.0%) reported their highest educational level as university (2–4 years). Table 1 summarizes participants’ current place of residence and highest level of education.

**Table 1.** Participants’ Current Place of Residence and Highest Level of Education.

Current place of residence	Frequency	Percent
Large-sized city	54	50.9
Medium-sized city	22	20.8
Smaller city	17	16.0
Rural area	13	12.3
Total	106	100.0
Highest level of education		
Not finished elementary school	2	1.9
Elementary school	11	10.4
Gymnasium—vocational preparation	8	7.5
Gymnasium—university preparation	19	17.9
University 2–4 years	35	33.0
University >4 years	26	24.6
Declined to answer	5	4.7
Total	106	100.0

### Procedure

This study was part of a larger research project entitled “Female Rape Victims: Quality of Initial Police and Medical Care Contact,” funded by the Swedish Crime Victim Compensation and Support Authority (Grant No: 3108/18). Other studies from the project include qualitative studies on the experiences of police meeting with victims and victims’ experiences of meeting with the police. The project was reviewed and approved by the regional ethical board in Gothenburg and by the Swedish Ethical Review Authority (Ref No: 883 18/2023–00934-02).

Participants were recruited partly through an advertisement distributed via support organizations (e.g., Storasyster and Unizon), and partly through posters and information hand-outs distributed at gynaecological emergency units across Sweden. These contained information about the purpose of the overall project and the questionnaire study, and emphasized that participation was voluntary and anonymous.

The inclusion criteria were that participants had to be women aged 18 years or older who had been raped and who had reported it to the police and/or had sought medical care after being raped. Participants who were interested in taking part in the study were encouraged to follow a link to the questionnaire. One respondent was under the age of 18, and her answers were excluded from analyses.

### Questionnaire

The questionnaire focused partly on experiences of reporting to police, and partly on experiences of seeking medical care after being raped. The material on seeking medical care will be analyzed in other publications.

The questionnaire was constructed by the first author, basing questions partly on previous Swedish and international research, and partly on findings from the previous qualitative studies in the project. Prior to the collection of data, the questionnaire was reviewed by a police officer and discussed in the research group, and minor adjustments were made. The questionnaire was available in both a Swedish version and an English version (back-translated).

Background questions included year and place of birth, current place of residence, highest level of education, and vulnerabilities (e.g., neuropsychiatric disabilities, experiences of selling sex/prostitution). Participants were then asked if they had reported being raped to police on more than one occasion. Participants who answered that they had, were asked to answer the following questions based on the most recent occasion where they had reported to police (to access the most recent memories). Background questions about the rape included what year the rape took place, the number of perpetrators, the sex of perpetrator/s, relationship to perpetrator/s, place of rape (e.g., outside, in the home), degree of physical violence, and whether participants had been drinking alcohol at the time of the rape. Participants who had not reported to the police were asked about their reasons for not reporting. Participants who had reported to the police were asked if the case had been dropped or taken to court. If the case had gone to court, they were asked whether the perpetrator/s had been penalized or acquitted, followed by questions on processing times.

Participants who had been interviewed by the police on more than one occasion and/or had met with more than one officer were asked to answer the following questions based on the encounter that had made the strongest impression on them—positive or negative. Questions then followed on the gender of the interviewing officer, and the level of perceived empathy, validation, and belief in the participants story that the interviewing officer had shown. After that, a series of questions focused on the questions asked during the police interview followed. Participants were asked if they had been asked about alcohol consumption, sexual behavior, consent, and physical resistance. If respondents stated that they had been asked one or more of these questions they were asked to what extent they perceived the specific questions as relevant, empathic, intrusive, and blaming/offensive. Participants were also asked how they perceived the questions in general, and whether the interviewing officer had explained her/his line of questioning. All participants (i.e., those who had and had not reported to the police) were asked about their level of trust in the legal system as a whole and in police work. All participants were also asked to rate how important different forms of justice were to them (e.g., being offered support, police empathy, and respect), and to what extent they perceived that they had received justice. Lastly, participants were offered contact information for national support organizations and for the first author, and space was given to comment on the answers given or on the questionnaire in general.

### Statistical Analysis

All responses were processed using IBM SPSS Statistics (Version 29). For specific analyses, the variable containing information about whether a participant's case had been tried in court was made binary (yes/no; with cases still pending coded as no). The year of rape was also coded as binary (before/after the latest Swedish legislation on rape). An index was created for quality of encounter with police, based on responses to three questions (min = 1, max = 5) on the perceived level of empathy (*M *= 2.99, *SD *= 1.42), validation (*M *= 2.89, *SD *= 1.51), and belief in participant's story (*M *= 3.35, *SD *= 1.41; index scores, *M *= 3.03, *SD *= 1.34). The Cronbach's alpha coefficient for the index was .914, indicating good internal consistency.

## Results

### Descriptive Findings

Most participants (56.6%) had been raped during the past 5 years: about half (50.9%) had been raped after the legislative changes, and about half (49.1%) were raped prior to the latest Swedish legislation on rape (2018). All participants (*N *= 106) had been raped by a man/men, and most participants (88.7%) reported a single perpetrator. Most participants (69.8%) reported that the rape took place in a home environment, and most participants (40.6%) stated their relationship to the perpetrator/s as a temporary acquaintance. Forty-six participants (44.7%) reported that they had been drinking alcohol at the time of the rape, while 57 participants (55.3%) reported that they had not. Most participants (51.9%) reported that moderate physical violence was used during the rape. Table 2 summarizes participants’ relation to perpetrator/s and the degree of physical violence used.

**Table 2.** Participants’ Relationship to Perpetrator/s and Degree of Physical Violence Used.

Relationship to perpetrator/s	Frequency	Percent
Temporary acquaintance	43	40.6
Unacquainted	16	15.1
Friend	13	12.3
Partner/cohabitant	12	11.2
Coworker	10	9.4
Boyfriend	6	5.8
Relative	3	2.8
Decline to answer	3	2.8
Total	106	100.0
Degree of physical violence used		
No physical violence	31	29.2
Moderate physical violence	55	51.9
Severe physical violence	15	14.2
Decline to answer	5	4.7
Total	106	100

Seventy-four participants (69.8) reported to the police. Fifteen participants (20.3%) indicated that their case had been tried in court (11 cases of which resulted in convictions, three in acquittals, and one still pending). Processing times for participants who had their case tried in court varied between less than 3 months (*n *= 4) and more than a year (*n *= 4). Forty-five participants (60.8%) reported that their case had been dropped without charge, and processing times for participants who had their case dropped without charge varied between <1 week (*n *= 5) and >1 year (*n *= 4). At the time of filling out the questionnaire, 14 participants (18.9%) were still waiting to find out if their case would make it to court.

The most commonly reported vulnerability at the time of the rape was experiencing longer periods of mental ill-health (54.5%) and neuropsychiatric disability (24.8%). Participants reported vulnerabilities at the time of the rape are summarized in Table 3 (multiple answers possible)*.*

**Table 3.** Participants’ Vulnerabilities at the Time of the Rape.

Vulnerability	Frequency	Percent
Neuropsychiatric disability	26	24.8
Longer periods of mental ill-health	55	54.5
Drug/alcohol abuse	9	8.6
Homelessness	2	1.9
Experience of selling sex/prostitution	18	17.6

### Reporting to the Police

Thirty-two participants (30.2%) did *not* report to the police. The most common reason for not reporting was “I didn’t think it would lead to prosecution and sentencing” (*n *= 20). Table 4 summarizes participants’ reasons for not reporting to the police (multiple answers possible).

**Table 4.** Participants’ Reason for Not Reporting to Police.

Reasons *not* to report to police	Frequency
I didn’t think it would lead to prosecution and sentencing	20
I was afraid that others would find out what happened	14
I had a negative view of how police treat victims of sexual violence	14
I was ashamed of what happened	13
I was afraid of the perpetrator/s	10
I was afraid of being accused of defamation	9
I didn’t feel like I needed to	4
None of the above	4

Exploring whether specific vulnerabilities were associated with participants’ reporting or not reporting to the police; independent samples *t*-tests showed no associations with longer periods of mental ill-health (*p *= .068), neuropsychiatric disability (*p *= .34), drug/alcohol abuse (*p *= .32), or experience of selling sex/prostitution (*p *= .36). Only two respondents reported being homeless at the time of the rape, so no test was executed for this group.

### Quality of Encounters With the Police

As hypothesized, an independent samples *t*-test showed that participants meeting with police after the latest Swedish rape legislation reported a higher quality (index) of encounter with the police (*M *= 3.49, *SD *= 1.25), than participants that met with police prior to the new legislation (*M *= 2.37, *SD *= 1.16); *t*(72) = 3.86, *p *< .001 (one-tailed), *d *= .92, 95% CI [1.69, 0.54]. Furthermore, in line with what was hypothesized, an independent samples *t*-test did not show any significant differences in reported quality of police encounters (index) between respondents who were interviewed by a female or male officer (*p *= .23).

With regards to the quality of police encounters (index) and different vulnerabilities; an independent samples *t*-test showed that participants reporting experience of selling sex/prostitution reported a lower quality in police encounters (*M *= 2.23, *SD *= 1.02) than participants who reported no such experiences (*M *= 3.30, *SD *= 41.31); *t*(69) = −2.74, *p *= .008 (two-tailed), *d *= .84, 95% CI [−1.84, −0.29]. Independent samples *t*-tests showed no significant differences in reported quality of police encounters (index) between participants with neuropsychiatric disability (*p *= .18), mental ill-health (*p *= .10), or drug/alcohol abuse (*p *= .12). Only two respondents reported being homeless at the time of the rape, and so no test was executed for this group.

### Questions Asked During Police Interview

Most participants perceived the general questions during police interviews as “not very relevant” (33.3%), “not at all empathic” (27.5%), “intrusive” to quite a high extent (36.0%), and as “victim blaming” to quite a high extent (24.3%). With regards to specific questions during police interviews; questions about sexual behavior were perceived as least relevant (48.8%) and questions about consent as most relevant (28.3%). Questions about sexual behavior were perceived as most intrusive (59.4%) and most blaming (31.1%). It should be noted that participants received different specific questions during police interviews, depending on their situation. Table 5 summarizes participants’ perceptions of general and specific questions during police interviews.

**Table 5.** Participants’ Perceptions of General and Specific Questions During Police Interviews.

	Not at all	Not very	Neither or	Quite	Very	
General questions (*n *= 69)					
Relevant	2 (2.9%)	23 (33.3%)	11 (10.4%)	22 (31.9%)	11 (15.9%)
Empathic	19 (27.5%)	18 (26.1%)	13 (18.8%)	11 (15.9%)	8 (11.6%)
Intrusive	11 (15.5%)	5 (7.0%)	14 (19.7%)	26 (36.0%)	15 (21.1%)
Blaming	16 (22.9)	9 (12.9%)	13 (18.6%)	17 (24.3%)	15 (21.4%)
Questions about alcohol consumption (*n = *45)					
Relevant	12 (25.5%)	11 (23.4%)	11 (23.4%)	4 (8.5%)	7 (14.9%)
Empathic	18 (40.0%)	11 (24.4%)	8 (17.8%)	3 (6.7%)	3 (6.7%)
Intrusive	7 (19.6%)	3 (6.5%)	8 (17.4%)	18 (39.1%)	8 (17.4%)
Blaming	10 (21.3%)	6 (12.8%)	7 (14.9%)	11 (23.4%)	13 (27.7%)
Questions about sexual behavior (*n *= 30)					
Relevant	15 (48.4%)	6 (19.4%)	5 (16.1%)	1 (3.3%)	3 (9.7%)
Empathic	17 (53.1%)	5 (15.6%)	6 (18.8%)	-	2 (6.3%)
Intrusive	2 (6.3%)	3 (9.4%)	2 (6.3%)	5 (15.6%)	19 (59.4%)
Blaming	4 (12.5%)	3 (9.4%)	5 (15.6%)	9 (28.1%)	10 (31.3%)
Questions about consent (*n *= 59)					
Relevant	9 (15.0%)	10 (16.7%)	9 (15.0%)	12 (20.0%)	17 (28.3%)
Empathic	16 (26.7%)	15 (25%)	11 (18.3%)	7 (11.7%)	7 (11.7%)
Intrusive	8 (13.1%)	7 (11.5%)	14 (23.0%)	12 (19.4%)	17 (27.9%)
Blaming	14 (23.3%)	6 (10.0%)	8 (13.3%)	12 (20.0%)	16 (26.7%)
Questions about physical resistance (*n *= 58)					
Relevant	8 (13.6%)	15 (25.4%)	16 (27.1%)	12 (20.3%)	7 (11.9%)
Empathic	14 (23.7%)	18 (30.5%)	15 (25.4%)	5 (8.5%)	4 (6.8%)
Intrusive	9 (15.0%)	4 (6.7%)	19 (31.7%)	15 (25.0%)	12 (20.0%)
Blaming	11 (18.3%)	4 (6.7%)	14 (23.3%)	17 (28.3%)	12 (20.0%)

Twenty-three participants (30.7%) reported that the interviewing officer had explained their line of questioning, while 43 participants (57.3%) reported that no explanation had been given (nine participants chose the “do not know” alternative). The relationships between how the general questions during police interviews were perceived and whether the interviewing officer explained her/his line of questioning (explaining) were investigated using Pearson product-moment correlation coefficients. Significant correlations were found between explaining and intrusiveness, *r *= .337, *n *= 75, *p *= .003 (two-tailed) and between explaining and blaming, *r *= −.296, *n *= 75, *p *= .010 (two-tailed), with explaining associated with a lower level of perceived intrusiveness and a lower level of perceived blaming. No correlations were found between explaining and perceived relevance (*p *= .47) or between explaining and perceived level of empathy (*p *= .07).

### Trust in the Swedish Legal System and in Swedish Police Work

According to the Swedish Crime Survey 2021, 55% of women in the general population reported a high level of trust (“quite high” or “very high”) in the legal system. In line with what was hypothesized, a chi-square goodness of fit test indicated a significant difference in the level of trust in the legal system between participants (22%) compared with the value of 55% that was obtained in the previous nationwide study, χ^2^ (1, *n *= 104) = 122.57, *p *< .001.

According to the Swedish Crime Survey 2021, 62% of women in the general population reported a high level of trust (“quite high” or “very high”) in police work. In line with what was hypothesized, a chi-square goodness of fit test indicated a significant difference in the level of trust in police work reported by participants in the present study (28%) compared to the value of 62% that was obtained in the previous nationwide study, χ^2^ (1, *n *= 101) = 70.36, *p *< .001.

The relationships between trust in the legal system, in police work, and ratings of quality of encounters with the police (index) were investigated using Pearson product-moment correlation coefficients. A significant correlation was found between quality of encounter and trust in the legal system *r *= .503, *n *= 72, *p *< .001 (one-tailed), with higher quality encounters associated with a higher level of trust in the legal system. A significant correlation was found between the quality of encounters and trust in police work, *r *= .732, *n *= 72, *p *< .001 (one-tailed), with higher quality encounters associated with a higher level of trust in police work. Independent samples *t*-tests indicated no significant differences between participants who did or did not report to the police regarding trust in the Swedish legal system (*p *= .48) or trust in police work (*p *= .27).

### Justice and Its Relationship With Quality of Police Encounters

**Figure 1.**
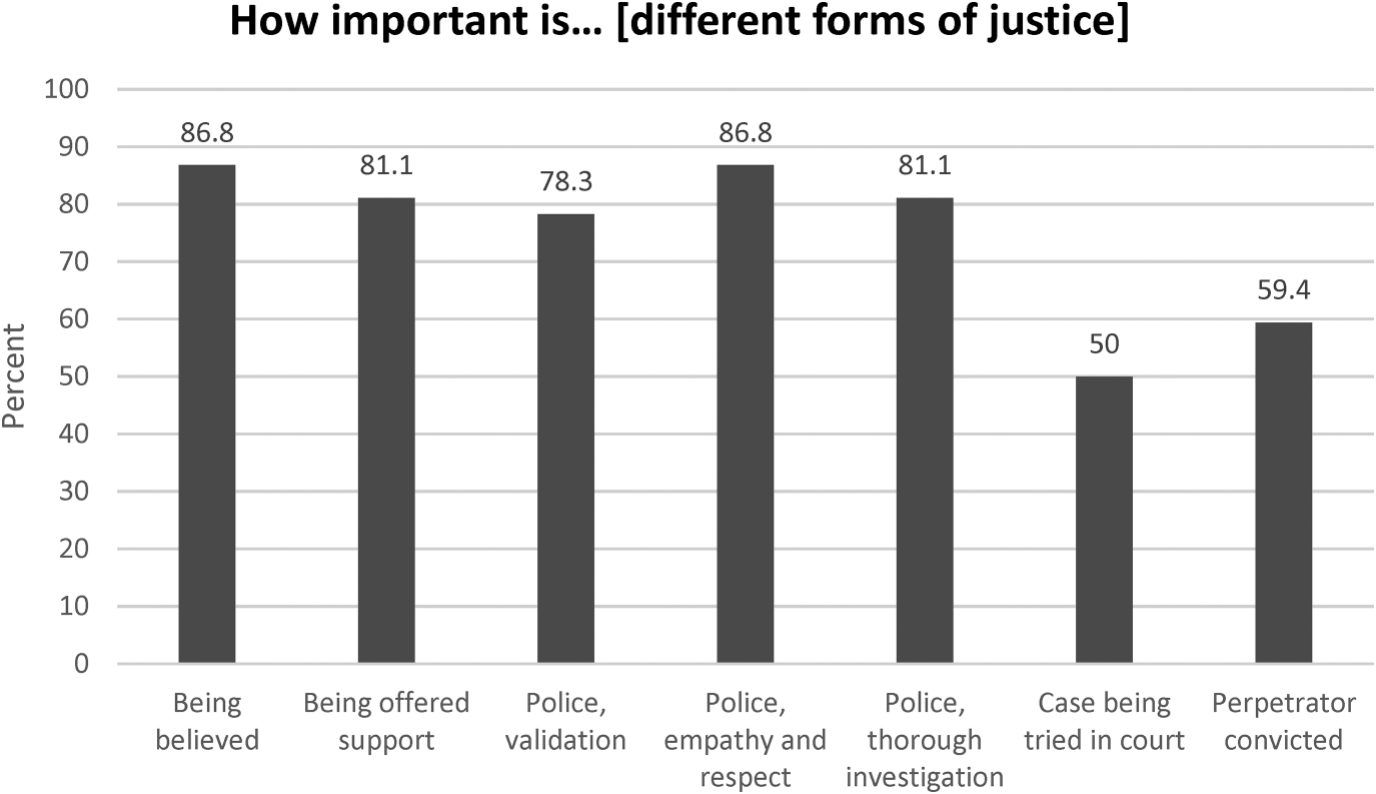
Participants’ views on justice (response alternative “very important form of justice”).

**Figure 2.**
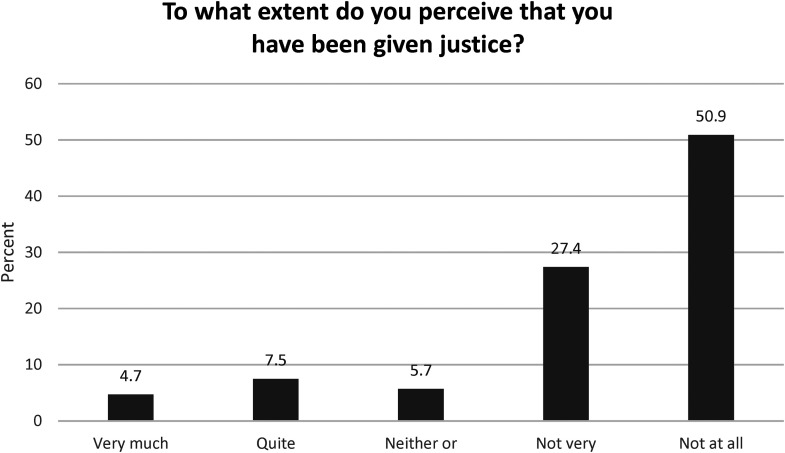
Participants’ perceptions of received justice.

Participants reported that feeling believed, being offered support, police validation, police empathy and respect, and police conducting a thorough investigation were important forms of justice. Although still important, having the case tried in court and the perpetrator being convicted were reported as being less important. Only 13 respondents reported that they had received justice. Figure 1 shows participants’ ratings of different forms of justice as “very important,” and Figure 2 shows participants’ perceptions of received justice.

An independent samples *t*-test showed that participants who reported to the police (*M *= 1.93, *SD *= 1.32) reported a higher level of received justice than participants who did not report (*M *= 1.34, *SD *= 0.60); *t*(101) = 2.39, *p *= .019 (two-tailed), *d *= .51 95% CI [0.10, 1.07].

A simple regression analysis showed that having the case tried in court explained a significant amount of the variance in the level of received justice, *F*(1, 74) = 48.56, *p *< .001, *R*^2^ = .403, *R^2^*_adjusted_ = .395. The regression coefficient (*B* = 2.08, 95% CI [1.48, 2.67]) indicated that having the case tried in court explained approximately 40% of the variance in received justice. Another simple regression analysis showed that quality of police encounters (index) explained a significant amount of the variance in level of received justice, *F*(1, 72) = 8.81, *p* = .004, *R*^2^ = .109, *R^2^*_adjusted_ = .097. The regression coefficient (*B* = .325, 95% CI [.11, .54]) indicated that higher quality police encounters explained approximately 10% of the variance in received justice.

## Discussion

Previous research has often focused on assessments of police attitudes regarding rape myths and attitudes toward victims. While these perspectives are important, Mourtgos et al. (2021) argue that assessing police behavior should be of primary importance. Furthermore, little attention has been paid to the views of the police among large populations of raped women (Lorenz, 2023). This discussion is focused on learning from the experiences of victims—victim's reporting, their need to tell, the need for trauma-informed police responses, for information and explanation to be given during questioning, and—ultimately—for justice.

### Victim Reporting and Their Need to Tell

Rape remains one of the most underreported violent crimes (Brookes-Hay, 2020). In Sweden, it is estimated that only one in five rapes are reported to the police (Holmberg & Lewenhagen, 2019). A similar picture has emerged in other European countries (Hohl & Stanko, 2015; Kelly, 2002), and in the United States (Brookes-Hay, 2020; Fisher et al., 2003). In this study, 67% of participants had reported to the police—a significantly higher percentage than in previous research of a similar nature. However, the invitation to participate stated that the questionnaire was aimed at women who had reported a rape to police and/or sought medical care after being raped. Consequently, women who had neither reported nor sought medical care may not have come forward or felt that they were unable to participate.

More than half of the participants in this study reported having experienced longer periods of mental ill-health and about one in four reported having been diagnosed with some form of neuropsychiatric disability. In addition, just under 18% of participants reported experiences of selling sex/prostitution. A study by Murphy-Oikonen et al. (2022) noted that certain vulnerabilities—including mental health issues, learning difficulties, being a sex worker or being an asylum seeker—were alarmingly common among victims of sexual offenses. They suggest that this is indicative of susceptibility to cumulative vulnerabilities in rape cases and that rape may be a significant risk for those with a broader ecology of overall vulnerability. Such findings, they argue, emphasize the need for trauma-informed policing as a response to sexual assault. They also propose that training for officers on both the stigma and occupational hazards surrounding such vulnerabilities is vital (Murphy-Oikonen et al., 2022). However, in this study, no specific vulnerabilities were associated with not reporting to the police. This contradicts the findings of McBride et al. (2020), who found that sex workers were less likely to report violence to the police. However, as a larger percentage of participants in this study had reported to the police it is hard to draw conclusions on reporting rates based on the present study.

In previous research, one often stated reason for low reporting rates is victims’ anticipation of negative treatment by the justice system (Patterson et al., 2009; Rich, 2019; Shaw et al., 2017). This reason was also commonly stated by participants in the present study, alongside participants’ being afraid that others would find out what had happened. The most common reason for not reporting, however, was participants not thinking that reporting would lead to prosecution and sentencing—indicating victims’ resignation and hopelessness. Although prosecution rates for rape charges in Sweden have increased by 75% since the new legislation (BRÅ, 2020), most rape charges are still dismissed (Holmberg & Lewenhagen, 2019). Victims’ fear that their reporting will not lead to trial may be based on knowledge of these facts. Furthermore, the finding that participants in this study reported that feeling believed, being offered support, police validation, police empathy and respect, and police conducting a thorough investigation where important forms of justice indicate that a victim's decision to report might be based on the hope of meeting an understanding officer, rather than on the expectation of receiving a positive judicial outcome. Hence, it could be argued that victims’ decisions to report may have less to do with successful conviction rates, and more to do with the possible benefits of empathetic police interactions, and the need for victims to tell their story.

Although not included in the main analysis, comments made in the questionnaire also provide insights. Forty-seven participants choose to leave comments, and these were largely about the importance of feeling believed, being offered support, and the need to be able to further elaborate on the specifics of the rape and how others had reacted to their story. These comments further indicate that victims feel a need to tell their story and emphasize the importance not only of police being willing to listen and understand but also the importance of other professionals and members of society, in general, being willing to do the same.

### Victims’ Perceptions of Police Interactions

In previous studies, the importance of police encounters has been stressed (Hopper et al., 2020), and victims report that being treated with dignity and respect by police is just as important to them as the legal outcome of their case (Jordan, 2015). Harmful responses on the part of the police can generate multiple negative outcomes, including exacerbating the stress and trauma experienced by victims (i.e., revictimization; Patterson, 2011), on victim willingness to engage and cooperate with the investigation (McKee et al., 2020), and can increase the risk of victim withdrawal (Mourtgos et al., 2021). They can also reduce the likelihood of a victim contacting the police again or reporting crimes in the future (Lorenz et al., 2021). In contrast, positive or supportive responses can be validating, reduce self-blame, and lead to victims seeking further help (Barkworth & Murphy, 2016; Lorenz, 2023).

Interactions perceived as negative are often characterized by officers being insensitive, rushing the victim, not allowing victims to steady themselves after emotionally exhausting questions, and questioning without building rapport (Greeson & Campbell, 2015). Factors contributing to positive outcomes include caring for the victim's emotions and well-being, being compassionate toward the victim's needs, and being personable with the victim by building rapport (Greeson & Campbell, 2015). Many victims—at least half of those who report to the police—experience negative responses or interactions (Ahrens et al., 2007). In this study, the average score on the index used to measure the quality of police encounters (based on perceived level of empathy, belief in the participant's story and validation) was 3.03. Given that the maximum score was 5, this suggests that, overall, encounters with the police tended to be viewed as more positive than negative, although were still a considerable way from being the best that they could have been. There was still a substantial proportion of the sample who rated their encounters with the police at the lower end of the index, suggesting that many still experience negative responses or interactions. It is important to note, too, that there were considerable variations in ratings of quality of encounters across the sample, with certain groups tending to report more negative experiences with the police than others. For example, those participants who reported experience of selling sex/prostitution gave index scores for their encounters with the police that were significantly lower than those given by participants who had no such previous experiences. Furthermore, while neuropsychiatric disability, mental ill-health, or drug/alcohol abuse were not found to significantly impact perceptions of police encounters here, there is reason to expect that there will be some variations in experience and quality of encounter as a result of negative stigmatization from police and law enforcement against certain victims or victim groups (e.g., McBride et al., 2020). Consequently, while things are improving—evidenced by the fact that those whose interactions with the police took place after the legislative changes reported a significantly higher quality of encounter than those whose cases were processed under the older legislation—there is clearly still a way to go until all victims’ experience police encounters that are empathetic, supportive, and facilitative.

The present research also explored a range of factors which may impact on perceived quality of police encounters, including the gender of the interviewing officer. Previous findings in relation to the benefits of victims of rape and sexual offenses being interviewed by female officers have been mixed. While some studies (e.g., Gregory & Lees, 1999; Temkin & Krahe, 2008) have suggested that female officers endorse fewer rape myths than their male counterparts, and thus tend to treat victims with more compassion, others find no significant impact of officer gender on victims’ perceptions or judgments of the encounter (e.g., Jordan, 2002). This was the case here; there was no significant difference in the reported quality of police encounters between respondents who were interviewed by a female or male officer, with index scores being similar for each. It may be that what is more important than the actual gender of the interviewing officer is offering victims’ a choice, allowing them some kind of agency. Rich (2019) proposes that a fundamental feature of an effective, trauma-informed approach includes offering victims’ options; for example, allowing them to choose to have a female detective conduct the interview (Chowdhury-Hawkins et al., 2008). In Sweden, there is no formal assignment of female officers to female victims; however, in previous studies, officers have described assigning women officers to women victims as “common sense” (Rudolfsson & Sinani, 2022, p. 434). Furthermore, in another previous Swedish study (Rudolfsson, 2023), victims describe how being offered the choice of having female officers respond sometimes resulted in less experienced officers being assigned to their cases. It is well established that the complexities of rape cases and the needs of victims demand a specialized response (Jordan, 2011). The results of this study, therefore, further highlight that the gender of the responding officer may be of less importance than their level of experience, specialized training, and the individual characteristics of showing empathy and support.

### Victims’ Need for Information During Questioning, Trust, and Need for Justice

Webster et al. (2021) discuss how interviews with victims of sexual offenses often contain inappropriate lines of questioning, and are not typically attentive to the specific needs of victims. This can have notable consequences; for example, Campbell and Raja (2005) found that the way in which victims were questioned directly impacted their perceptions of the interactions, with many stating that the experience had made them feel guilty, distrustful, depressed, and/or anxious, which was reflected in their unwillingness to disclose information. In contrast, where victims are treated sympathetically, with sensitive questioning, this can help produce stronger victim statements which can be used to build stronger cases for prosecution (Patterson, 2011).

Patterson (2011) observes that it is important to examine the nature and quality of interviews from the victims’ viewpoint in order to better understand the dynamic interplay between interviewee and interviewer, which may affect the quality of the case that is put together. The type and manner of questioning that the participants in the present study experienced was consequently a focal point of the research, to try and ascertain the degree to which this impacted overall ratings of police encounters and experiences. Results show that where officers explained their line of questioning the perceived intrusiveness of the questioning was lower, as was the level of perceived victim-blaming. However, the majority of participants reported that no explanation about the line of questioning was given in their encounter with the police. Most participants reported that they perceived the general questions asked during police interviews to be not very relevant, to be not at all emphatic, to be intrusive, and to demonstrate victim-blaming. Questions regarding sexual behavior were perceived as the most intrusive and demonstrated the highest degree of blaming. These findings accord with those reported previously, with other samples from other parts of the world (e.g., Johnson, 2015).

Victim accounts are crucial to the success of a case, and research has shown that giving investigating officers intensive, specialized training can improve their interviewing skills, improve both the quality and quantity of information obtained through interviews and result in a better experience for victims (Rich, 2019). However, there is often scant police training on victim interviewing, because the interrogation and apprehension of suspects are viewed as a priority (Horvath et al., 2011). From a victim perspective, previous research demonstrates that stress reactions are easily aroused in traumatized individuals (Foa et al., 2019) and, further, that situations perceived as unpredictable increase victims’ stress levels, while predictability decreases stress (Sapolsky, 2004). Previous research also demonstrates the importance of police training to identify and respond appropriately to victims’ potential trauma reactions (Spohn, 2020). Further, it highlights how important a victim’s feeling of safety during the interview is to their ability to describe the assault (Rich, 2019). The safer a victim feels during interviews, the more information they will access and report, and—in turn—the more accurate the statements they give will be (Hopper et al., 2020). Consequently, the results of this study further indicate the importance of officers’ trauma-informed training, and the value of officers explaining their line of questioning to create a less stressful environment for the victim and reduce the risk of the victim's feeling doubted and blamed, with the further benefit of producing stronger victim statements.

In previous research, Sweden ranks highly regarding the general population's trust in the police (Statista Research Department, 2022). In this study, however, victims of rape reported a significantly lower trust in the legal system, and in police work specifically, than women in the general Swedish population. In previous research, demographic factors such as socioeconomic status and race have been found to influence the views of the police (Lorenz, 2023). Furthermore, both negative direct interactions and vicarious experiences (e.g., those of friends and seen in the media; Lorenz, 2023; Rosenbaum et al., 2005) have been found to be associated with negative views of the police.

In this study, participants reported that feeling believed and the police showing empathy and respect were the most important forms of justice; however, more than half of the participants reported that they had not received any justice. This study also found that victims’ having their cases tried in court explained a significant amount of the variance in received justice; however, quality in police encounters also explained participants’ perceptions of received justice to a significant degree. In line with this, procedural justice is defined as victims’ being treated with dignity and respect and feeling heard throughout the process (Hohl et al., 2022), and perceptions of procedural justice in police encounters have been found to influence victims’ self-worth, self-value, trust, and willingness to participate in the investigation (Hohl et al., 2022), indicating the judicial and psychological values of police interactions. Consequently, procedural justice is a policing strategy built on the elements of voice, respect, neutrality, trustworthiness, and empathy (Rosenbaum & Lawrence, 2017). From this perspective, the findings of this study that only 13 participants reported that they had received justice is a call for action.

### Methodological Reflections and Limitations

Previous research calls for more creative ways to measure the demeanor of police officers in their encounters with victims of rape (Mourtgos et al., 2021). We, therefore, created a questionnaire based partly on previous research, partly on results from our own qualitative studies focusing on police and women who have been raped. Creating our own questionnaire allowed us to use issues not previously studied, for example, how specific questions during police interviews were perceived. Although the questionnaire was reviewed by other researchers and by a police officer, it should, however, be noted that the questions included have not been validated in previous research.

The focus on female victims in this project was partly due to the specifics of female gynecological examinations, and partly due to the acknowledgment of the fact that women are more often victims of sexual violence (BRÅ, 2023; WHO, 2021). Participants were recruited through advertisements at nonprofit support organizations and gynecological emergency units in Sweden. This method of recruitment has previously been described as suitable for reaching those affected by a specific area of interest (SBU, 2016).

Both the information and the questionnaire were available in Swedish and in English. However, it should be noted that only one participant answered the English version, possibly indicating a bias in recruitment favoring Swedish-speaking women. Furthermore, previous research notes that victims label their experiences in different ways, affecting their likelihood of reporting (Canan et al., 2023). In a previous qualitative study (Rudolfsson, 2023), women described how they had been made to understand that what happened was actually rape in interactions with friends and relatives; preferring themselves, at first, to talk about it in lesser terms. Consequently, as the information sheet for this study stated that we were looking for women who had been raped, this may have biased our sample, with some women not feeling invited to participate in the same way.

Participants in this study reported differing lengths of time having passed since the rape took place. Not only had participants had varied amounts of time to process what had happened, but it is also reasonable to assume that memories fade or change as more time passes (de Oliviera et al., 2021). This should not be interpreted as participants being less accurate in their memories of events, as previous research on memory and trauma concludes that a victim’s memory can improve over time and that there is no scientific basis for the assumption that a victim’s later memories are less credible (Hopper et al., 2020). Furthermore, participants had reported to police under different Swedish laws. The decision not to restrict the timeframe for participation was based on ethical reasons, acknowledging victims’ need for forums where they can share their experiences. This decision also allowed us to compare perceptions of reporting before and after the latest legislative amendment.

The dependent variable on received justice was not normally distributed (skewness = 1.314). However, Hair and Alamer (2022) concludes that a skewness value of between –2 and +2 is generally considered acceptable. This study asked for victims’ reasons *not to* report. Future studies could benefit from also including questions on reasons why victims *do* report.

### Practical Implications and Recommendations

The biggest sources of attrition in rape cases are within the police investigation stage and relate to both decisions by police officers and victims (Murphy-Oikonen et al., 2022). Findings from the present study demonstrate the importance of police explaining lines of questioning, giving reasons as to why certain kinds of questions are being asked and how they are relevant. The findings of this study also demonstrate the importance of making sure that, where possible, the most experienced officer(s) are utilized, particularly for conducting interviews with victims.

Improving training for law enforcement—particularly with regard to interviewing—should be a priority (Patterson, 2011). Previous research demonstrates that specialized training programs aimed at teaching law enforcement how to build rapport with victims are successful. Any such training implemented must be properly evaluated, to ensure that it is achieving what it sets out to do (Murphy-Oikonen et al., 2022). More broadly, a more tailored approach to rape victims is needed; one which considers vulnerabilities and specialized needs. Such trauma-informed approaches are recognized as “best practices” but have not been widely disseminated or integrated into everyday police work (Rich, 2019). One way in which these might be implemented is through having specialist investigators and specialist investigative practices for rape and sexual offenses. In relation to a range of measures, police specialist rape investigation units have been found to outperform nonspecialist units both in Sweden (Backhans et al., 2021) and in the United Kingdom (Rumney et al., 2020). Consequently, a need for more such units is indicated.

### Concluding Remark

The current research takes steps toward understanding factors which may influence victim's decisions; in doing so, it provides insights which might help inform policy and practice developments, both in Sweden and more generally, to help narrow the justice gap currently experienced by many victims of sexual offenses. By exploring the influence of the experiences of victims of sexual offenses on their views of the police, their perspectives can be considered in decisions regarding funding and reform that best represent victims’ needs and interests (Lorenz, 2023). We hope that the present study will help in this regard, and we wish to thank the participants for sharing their experiences with us.
